# Intra-host dynamics of co-infecting parasite genotypes in asymptomatic malaria patients

**DOI:** 10.1016/j.meegid.2018.08.018

**Published:** 2018-11

**Authors:** Standwell C. Nkhoma, Rachel L. Banda, Stanley Khoswe, Tamika J. Dzoole-Mwale, Stephen A. Ward

**Affiliations:** a Malawi-Liverpool-Wellcome Trust Clinical Research Programme, University of Malawi College of Medicine, Blantyre, Malawi; b Liverpool School of Tropical Medicine, Pembroke Place, Liverpool L3 5QA, UK; c Wellcome Trust-Liverpool-Glasgow Centre for Global Health Research, 70 Pembroke Place, Liverpool L69 3GF, UK

**Keywords:** *Plasmodium falciparum*, Single Nucleotide Polymorphism, Superinfection, Parasite co-transmission, Within-host parasite diversity, Asymptomatic malaria

## Abstract

Malaria-infected individuals often harbor mixtures of genetically distinct parasite genotypes. We studied intra-host dynamics of parasite genotypes co-infecting asymptomatic adults in an area of intense malaria transmission in Chikhwawa, Malawi. Serial blood samples (5 ml) were collected over seven consecutive days from 25 adults with asymptomatic *Plasmodium falciparum* malaria and analyzed to determine whether a single peripheral blood sample accurately captures within-host parasite diversity. Blood samples from three of the participants were also analyzed by limiting dilution cloning and SNP genotyping of the parasite clones isolated to examine both the number and relatedness of co-infecting parasite haplotypes. We observed rapid turnover of co-infecting parasite genotypes in 88% of the individuals sampled (*n* = 22) such that the genetic composition of parasites infecting these individuals changed dramatically over the course of seven days of follow up. Nineteen of the 25 individuals sampled (76%) carried multiple parasite genotypes at baseline. Analysis of serial blood samples from three of the individuals revealed that they harbored 6, 12 and 17 distinct parasite haplotypes respectively. Approximately 70% of parasite haplotypes recovered from the three extensively sampled individuals were unrelated (proportion of shared alleles <83.3%) and were deemed to have primarily arisen from superinfection (inoculation of unrelated parasite haplotypes through multiple mosquito bites). The rest were related at the half-sib level or greater and were deemed to have been inoculated into individual human hosts via parasite co-transmission from single mosquito bites. These findings add further to the growing weight of evidence indicating that a single blood sample poorly captures within-host parasite diversity and underscore the importance of repeated blood sampling to accurately capture within-host parasite ecology. Our data also demonstrate a more pronounced role for parasite co-transmission in generating within-host parasite diversity in high transmission settings than previously assumed. Taken together, these findings have important implications for understanding the evolution of drug resistance, malaria transmission, parasite virulence, allocation of gametocyte sex ratios and acquisition of malaria immunity.

## Abstract (author highlights)

• We examined if a single blood sample from a malaria-infected individual accurately captures within-host parasite diversity
• We also examined patterns of kinship between co-infecting parasite haplotypes to infer malaria transmission dynamics
• A single sample poorly captured within-host parasite diversity indicating the need for repeat sampling of infections
• Single mosquito bites played a key role in generating within-host parasite diversity even in this high transmission area

## 1 Introduction

Malaria remains a major global health problem and one of the leading causes of morbidity and mortality especially in tropical regions of the world. There were 216 million malaria cases and 445,000 malaria-related deaths in 2016 alone (World Health Organization, 2017). Compared with estimates for the year 2000, these figures represent a 41% and 62% decline in malaria cases and malaria-related deaths respectively. Although these statistics show remarkable progress in global malaria fight, they represent only subtle gains for sub-Saharan Africa where most (>80%) of the global malaria burden is concentrated. The intransigence of malaria in the face of concerted and sustained control efforts in sub-Saharan Africa calls for renewed political will to fight malaria as well as new interventions and novel delivery systems for existing interventions to maximize their impact on reducing malaria burden.

Malaria is caused by an obligately sexual protozoan parasite of the genus *Plasmodium* and is transmitted to human beings via bites from *Anopheles* mosquitoes. For most of its life cycle, the malaria parasite exists as a haploid organism except during a brief diploid phase when male and female gametocytes (sexual stages of the parasite) fuse to form a zygote. Genetic recombination occurs only during the sexual phase in the mosquito and generates new parasite haplotypes as genes are reshuffled and re-assorted at this stage. For novel parasite diversity to be generated, the mosquito must ingest a human blood meal containing a mixture of genetically distinct male and female gametocytes. If a mosquito takes a blood meal containing genetically identical gametocytes, self-fertilization (selfing) will occur. This process will not generate new parasite genetic diversity because the resultant progeny will bear the same haplotype as their parents. The level of outbreeding depends on the proportion of individuals harboring multiple parasite genotypes and strongly correlates with *P. falciparum* prevalence (Anderson et al., 2000; Fola et al., 2017) but poorly with *P. vivax* prevalence (Fola et al., 2017). While ingestion of a human blood meal containing genetically diverse parasites is required to generate new parasite diversity in the mosquito vector, within-host parasite diversity in humans can arise from two main processes. First, sequential bites from multiple malaria-infected mosquitoes can introduce unrelated parasites into a single human host. Second, mixtures of genetically related parasites previously generated by recombination in the mosquito can be inoculated into individual human hosts through single mosquito bites. These processes are referred to as “superinfection” and “parasite co-transmission” respectively (Nkhoma et al., 2012; Alizon, 2013; Wong et al., 2017). The process that predominates in generating within-host parasite diversity can be inferred from patterns of kinship between co-infecting parasite haplotypes.

Infection by malaria parasites does not always translate to a full-blown clinical disease with classical malaria symptoms such as headache and fever chills. Some residents of malaria-endemic areas do not show any clinical symptoms of malaria despite being infected (Bousema et al., 2014; Chen et al., 2016). These individuals may remain asymptomatic for weeks or even months following infection depending on several factors such as their level of premunition and the presence of concomitant infections (Doolan et al., 2009; Chen et al., 2016). Adults with asymptomatic malaria are a unique group of malaria research participants because they can be left untreated for a few days to study the natural course of malaria infections. This is the case because asymptomatic adults have a reduced risk of developing severe anaemia or other forms of complicated malaria requiring hospitalization. Therefore, asymptomatic adults can provide a unique window into within-host dynamics of naturally occurring and untreated malaria infections.

The purpose of this study was to determine how accurately a single peripheral blood sample taken from a malaria-infected individual captures within-host parasite diversity, and to characterize the within-host population structure of malaria parasites in asymptomatic adults. While some findings support the notion that a single blood sample contains the full spectrum of parasite genotypes present in a malaria-infected individual (Missinou et al., 2004; Dembo et al., 2006; Waltmann et al., 2018), others suggest that it harbors only a subset of constituent parasite genotypes (Daubersies et al., 1996; Farnert et al., 1997; Kamwendo et al., 2002; Farnert, 2008; Farnert et al., 2008). These discrepant findings may be due to the low resolution of msp-1 and msp-2 genotyping used to fingerprint malaria infections in previous studies. Limited discriminatory power of msp-1 and msp-2 genotyping may result in failure to capture minority parasite variants and could lead to the detection of only a subset of parasite genotypes present within the individual. Apart from examining whether single snapshots of peripheral blood adequately capture within-host malaria parasite diversity, this study focused on elucidating the within-host population structure of malaria parasites. Such type of studies can aid modelling of important malaria traits by providing empirical data for testing, refining and validating model assumptions. Methods for estimating inbreeding coefficients and the mean number of parasite haplotypes within malaria-infected blood samples assume that multiple-haplotype infections (MHIs) predominantly arise from superinfection such that their component parasite haplotypes are genetically unrelated and independent of each other (Hill and Babiker, 1995; Hill et al., 1995; Ross et al., 2012). Similarly, models and studies of malaria transmission, parasite virulence, drug resistance, and gametocyte sex ratio allocation generally assume independence and non-relatedness of co-infecting parasite haplotypes (Nowak and May, 1994; Reece et al., 2008; Antao and Hastings, 2011; Alizon et al., 2013; Kada and Lion, 2015). However, genetic analysis of MHIs from Blantyre, Malawi, where individuals experience an average of 2.8 episodes of symptomatic malaria per year (Sulo et al., 2002) revealed that MHIs predominantly contain related and non-independent parasite haplotypes (Nkhoma et al., 2012). In the neighboring Chikhwawa District, individuals encounter ~183 infectious bites per person per year (Mzilahowa et al., 2012). Because of higher levels of malaria transmission in Chikhwawa compared to Blantyre, we might expect patterns of within-host parasite relatedness generated through superinfection to predominate over those attributable to parasite co-transmission.

To determine whether a single blood sample accurately captures within-host parasite diversity, we genotyped serial blood samples from 25 asymptomatic adults, and compared parasite DNA fingerprint profiles of serial blood samples from the same individual. If a single blood sample accurately captures within-host parasite diversity, we would expect consecutive blood samples from the same individual to have an identical parasite DNA fingerprint profile. To examine relatedness amongst co-infecting parasite genotypes, we isolated individual parasite haplotypes from MHIs by dilution cloning (Rosario, 1981; Nkhoma et al., 2012) and used allele-sharing patterns to infer kinship. The expectation is that parasite haplotypes introduced by superinfection would be predominantly unrelated while those generated by parasite co-transmission would be mostly related.

## 2 Materials and methods

### 2.1 Setting up the study and sampling asymptomatic malaria infections

Prior to setting up the study in communities that we sampled, we held a series of community engagement meetings with key stakeholders to explain the purpose of our study, how communities will be involved and potential risks and benefits for participating in this study. These fora also provided an opportunity for stakeholders to raise any questions or concerns they had about the study. The study commenced following approval from chiefs in the catchment area, Chikhwawa District Health Office, College of Medicine Research and Ethics Committee (Protocol # P.02/13/1529) and Liverpool School of Tropical Medicine Research Ethics Committee (Protocol # 14.45RS). The catchment area of our study was ~ 64km^2^ in size and encompassed communities around Belo Health Centre. To identify eligible study participants, 82 adults (18–45 years old; 39 males and 43 females) without symptoms of malaria or any other known disease were screened for malaria parasites using the histidine rich protein II-based rapid diagnostic tests (RDTs) following the initial consenting process. Twenty-seven RDT-positive individuals were requested to provide additional written informed consent to participate in the study of which 25 (93%) agreed. During participant enrollment, we avoided recruiting members of the same or nearby households to preclude a within-host population structure driven by focal malaria transmission (Smith et al., 2017). Serial blood samples (5 ml) were collected over seven consecutive days from 25 consenting adults with positive RDTs. Blood samples were collected in Acid Citrate Dextrose tubes (BD, UK), and transported in an ice-cold container to the laboratory in Blantyre where sample processing and parasite genotyping were performed. Half of each blood sample was washed with incomplete RPMI-1640 media (Sigma-Aldrich, UK) and cryopreserved in Glycerolyte 57 Solution (Fenwal, Lake Zurich, IL, USA). Malaria parasites used in dilution cloning experiments were grown and culture-adapted from this sample. The second half of each sample was passed through a CF11 filtration column to deplete human leucocytes (Venkatesan et al., 2012). High quality parasite DNA was extracted from this sample and used in genotyping experiments. Enrolled study participants were carefully monitored by our clinical management team every day for any symptoms of malaria or other known disease. If symptoms of malaria or any other known disease did not appear, study participants were left untreated until the last day of follow up (day 7) when standard malaria treatment (Artemether-Lumefantrine) was administered to each study participant upon exiting the study. We assumed no new infections occur during follow up because newly inoculated sporozoites take at least a week to undergo full development in the liver and breakthrough the bloodstream to initiate a blood-stage infection (Soulard et al., 2015).

### 2.2 Parasite DNA extraction and genotyping

Parasite DNA was extracted from all serial blood samples from each of the 25 participants using DNA Mini Kits (Qiagen, UK). Each parasite DNA sample was genotyped using the 24-SNP Molecular Barcode Assay (Daniels et al., 2008) to determine the DNA fingerprint of constituent parasites. Parasite DNA samples from three of the 25 individuals were also genotyped at msp1 and msp-2 loci as described previously (Snounou et al., 1999) to obtain comparable fingerprint profiles. Parasite DNA samples from *P. falciparum* laboratory strains 3D7, K1, W2, DD2, HB3 and R033 obtained from MR4 (Manassas, VA) were included in genotyping runs as positive controls. Each genotyping run also included a negative water control. Briefly, 2.95 μl of nuclease-free water was mixed with 0.05 μl of the 40× Taqman SNP assay and 5 μl of the TaqMan Universal PCR Master Mix (Applied Biosystems Catalogue # 4364343) in each well of a 96-well real-time PCR plate pre-loaded with 2 μl (10 ng) of each parasite DNA sample. Details of the 24 SNPs genotyped including their corresponding primer and probe sequences were as in the original methodology paper (Daniels et al., 2008). Samples were amplified on the StepOne real-time PCR instrument (Applied Biosystems, USA) and results were analyzed using Applied Biosystem's proprietary Allelic Discrimination Software. Where the Allelic Discrimination Software failed to provide genotype calls directly, we manually assigned allelic calls to the samples by examining both their amplification and multi-component plots.

### 2.3 Resolving whether a single peripheral blood sample accurately captures within-host malaria parasite diversity

We performed two analyses to determine whether a single peripheral blood sample contains all parasite genotypes/haplotypes present in a malaria-infected individual. First, we compared parasite DNA fingerprint profiles of serial blood samples from the same individual. Second, we subjected serial blood samples from three participants to limiting dilution cloning and genotyped clones isolated to determine both the number and relatedness of parasite haplotypes in each blood sample. We would expect serial blood samples from the same individual to show identical parasite DNA fingerprints or bear the same array of parasite haplotypes if a single blood sample accurately captures total within-host parasite diversity.

### 2.4 Dilution cloning of a subset of asymptomatic infections

Serial blood samples from three asymptomatic individuals were cloned by limiting dilution (Rosario, 1981; Nkhoma et al., 2012) to isolate individual parasite clones. Because parasite densities in these individuals were below detection by light microscopy, parasites in all blood samples were cultured for ~ two weeks prior to cloning. To isolate single erythrocytes each infected with exactly one parasite, 100 μl of the parasite culture containing approximately five parasitized cells per ml of complete RPMI-1640 media was inoculated into each well of a sterile 96-well plate. The plate was incubated at 37 °C in a sealed gas chamber flushed with a 93% N_2_, 4% O_2_ and 3% CO_2_ gas mixture. On day 21 following cloning, parasite-positive wells were identified by detecting parasite DNA in each well using a DNA-labelling dye, Sybr Green 1 (Johnson et al., 2007). Briefly, 100 μl Lysis Buffer/Sybr Green 1 solution prepared by mixing 0.2 μl of 1000× Sybr Green 1 solution and 1000 μl of lysis buffer (20 mM Tris at a pH of 7.5, 5 mM EDTA, 0.008% *w*/*v* saponin and 0.08% *v*/v Triton X-100) was added to 100 μl of cells re-suspended from each well. The detection plate was incubated at room temperature for ~45 min in the dark and read at excitation and emission wavelengths of 485 nm and 530 nm respectively using the FLx800 Multi-Detection Microplate Reader (BioTek Instruments Inc., USA). Parasite-positive wells were identified as those with elevated fluorescence intensity relative to the background and were confirmed by light microscopy of blood films prepared from resuspended cells. Parasite clones isolated from each serial blood sample were genotyped using the 24-SNP Molecular Barcode Assay (Daniels et al., 2008) to determine both the number and relatedness of constituent parasite haplotypes.

### 2.5 Assessment of cloning efficiency

To assess how efficiently parasite diversity in the original infection was sampled, the number of wells containing clonal parasite lineages was compared with that expected assuming a Poisson distribution of parasite-positive wells in the 96-well cloning plate. Its probability mass function was generalized as:*p*(*k* = *n*) = [*λ*^*k*^*e*^−*λ*^]/*k*! where *p* is the probability that a single well is inoculated with exactly *k* parasitized erythrocytes (*k* ≥ 0), *λ* is the number of parasite-infected cells in the inoculum used to seed the wells (*λ* > 0) and *e* is Euler's number. For example, the probability that each well was seeded with exactly one parasitized erythrocyte is given by *p*(*k* = 1). We used the incidence ratio test implemented in STATA version 11.0 (College Station, TX) to determine if there were significant differences between the numbers of wells containing clonally derived parasites following dilution cloning of any two blood samples.

### 2.6 Resolving the number and relatedness of parasite haplotypes within patients

SNP data for parasite clones isolated from serial blood samples from three participants were used to determine both the number and relatedness of parasite haplotypes within patients. Parasite clones with ≥2 heterozygous SNP calls were considered multiclonal (Sisya et al., 2015) and were excluded from phylogenetic analyses because parasite haplotypes cannot be accurately reconstructed. Those with less than two heterozygous base calls were deemed monoclonal (single-haplotype infections). We used a threshold of ≥2 heterozygous SNPs to distinguish between single-haplotype and multiple-haplotype infections because one random SNP out of the 24 SNPs genotyped is occasionally incorrectly scored as mixed (heterozygous) even in well-characterized single-haplotype infections (Sisya et al., 2015). SNP data for single-haplotype infections were used to construct the UPGMA tree to describe relationships between parasite haplotypes recovered from the same or different infections. The tree was constructed with PHYLIP (http://evolution.genetics.washington.edu/phylip.html), using the genetic distance 1-*ps*, where *ps* is the proportion of alleles shared between parasites. To define the degree of relatedness between parasite haplotypes, we simulated allele-sharing expected for genetically unrelated parasites, half-sib parasites, full-sib parasites and parasites derived from inbreeding between full-sib parasites as described previously (Anderson et al., 2010; Nkhoma et al., 2012). These simulations were performed using SNP allele frequencies in single-haplotype infections only. We then assigned relatedness classes to haplotype relationships observed by fitting their observed pairwise allele-sharing into the simulated frequency distribution of pairwise allele-sharing expected of parasites in different relatedness categories. Because allele-sharing distributions for the different relatedness classes showed some overlap, the upper confidence limit for pairwise allele-sharing expected for unrelated parasites was used as a threshold for defining related and unrelated parasite haplotypes. Similarly, the upper confidence limit for pairwise allele-sharing expected for full-sib parasites was used as a cut-off for differentiating full-sibs and parasites more related than full-sibs.

## 3 Results

### 3.1 Patient recruitment and follow up success rate

Out of 82 healthy adults screened for malaria using RDTs, 33% (*n* = 27) were positive for *Plasmodium falciparum* parasites. Of the 27 parasite-positive individuals, 25 agreed to provide serial blood samples for use by this study while two refused and were immediately withdrawn from the study. Of the 25 fully consenting study participants, 24 completed all the study-related procedures including providing all the requested serial blood samples. However, participant MW11 withdrew from the study on day four of follow up citing concerns over repeated blood draws.

### 3.2 Parasite DNA fingerprinting of serial blood samples from individual participants

**Fig. 1:**
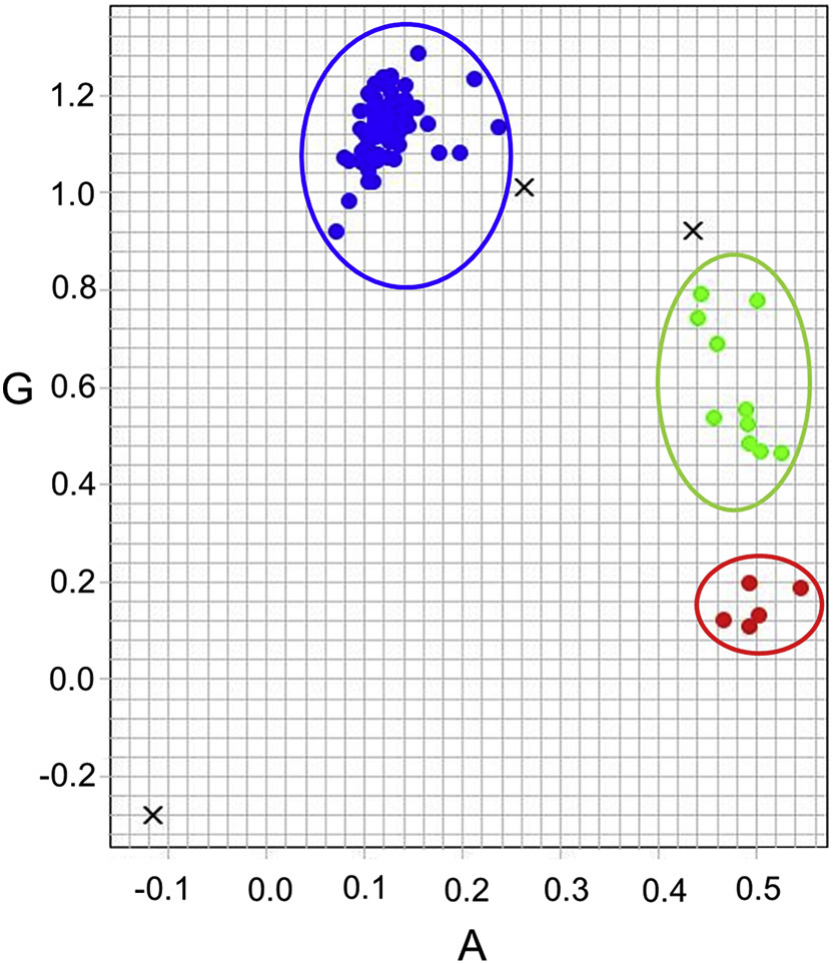
SNP genotyping. Shown here is a typical allelic discrimination plot with genotype results for the A/G SNP on chromosome 7 and position 000277104 of the *P. falciparum* genome (PlasmoDB version 5.0). Axes “A” and “G” represent fluorescence intensities for the “A” and “G” alleles in the samples genotyped. Each dot in the plot represents a normalized fluorescence intensity signal for each sample and provides the genotype call for the sample. Red and blue clusters contain samples that are homozygous for the “A” and “G” alleles respectively while the green middle cluster contains samples that are heterozygous for both alleles (i.e. multiclonal samples). The letter “X” in any area of the plot represents a sample without a SNP genotype call (i.e. a genotyping failure). (For interpretation of the references to colour in this figure legend, the reader is referred to the web version of this article.)

Serial blood samples from 25 asymptomatic adults were successfully genotyped using the 24-SNP Molecular Barcode Assay (Fig. 1, Supplementary Table S1). Nineteen of the 25 individuals sampled (76%) carried multiple parasite genotypes at baseline. Identical parasite DNA fingerprint profiles were observed in serial blood samples from each of the participants MW3, MW4 and MW6 (Supplementary Table S1). However, consecutive blood samples from 22 other participants had different parasite DNA fingerprints (Supplementary Table S1). Of the 174 serial blood samples genotyped, 33 carried zero or one heterozygous SNPs and were deemed monoclonal. Msp-1 and msp-2 genotyping of serial blood samples from three of the 25 participants (MW1, MW2 and MW3) confirmed the presence of identical parasite genotypes in consecutive blood samples obtained from participant MW3 but a rapid turnover of co-infecting parasite genotypes in serial blood samples taken from participants MW1 and MW2 (Supplementary Fig. S1).

### 3.3 Dilution cloning of infections and SNP genotyping of parasite clones isolated

**Fig. 2:**
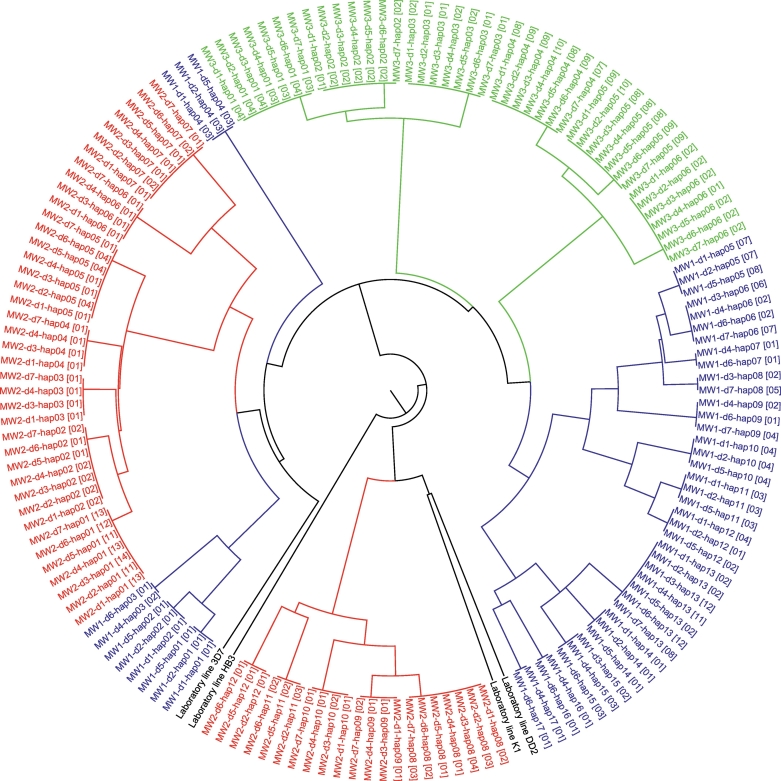
Within-host parasite relatedness and dynamics. Parasite haplotypes from the same patient are shown using the same colour scheme in red, blue or green while laboratory controls are shown in black. MW1, MW2 and MW3 are patient identifiers, d1 to d7 represent the seven different days of blood sampling while hap01 to hap17 are designations for parasite haplotypes recovered from each patient. For example, MW3-d1-hap01 [04] denotes parasite haplotype 1 recovered from patient MW3 on day 1 of blood sampling. The number, 04, in square brackets denotes the number of parasite clones bearing this haplotype. Some of the parasite haplotypes from the same patient cluster together on the tree while others are diverged from such clusters. Up to 17 parasite haplotypes (shown in blue) were isolated from a single patient. Data show a rapid temporal change in the genetic composition of parasites infecting patients MW1 and MW2 but not MW3. Data also show patterns of parasite co-transmission superimposed on those generated by superinfection in the same patients. (For interpretation of the references to colour in this figure legend, the reader is referred to the web version of this article.)

Dilution cloning of serial blood samples from patients MW1, MW2 and MW3 yielded 23 to 29 parasite-positive wells each containing a clonal population of parasites. The probability that each well was inoculated with a singly infected erythrocyte is given by *p*(*k* = 1) and equals exp.(−0.50)*(0.50^1)/fact(1) = 0.30. Since 96 wells were seeded, we expected that 29 random wells in each dilution cloning plate would contain parasites derived from singly-infected erythrocytes. SNP genotyping showed that 22 of 179, 8 of 171 and 3 of 185 parasite clones from patients MW1, MW2 and MW3 had ≥2 heterozygous SNP calls respectively (Supplementary Table S2) and were considered to harbor multiple parasite haplotypes. Phylogenetic analysis demonstrated that each of the three study participants MW1, MW2 and MW3 carried 17, 12 and 6 parasite haplotypes respectively (Fig. 2).

### 3.4 Within-host dynamics of parasite haplotypes

**Fig. 3:**
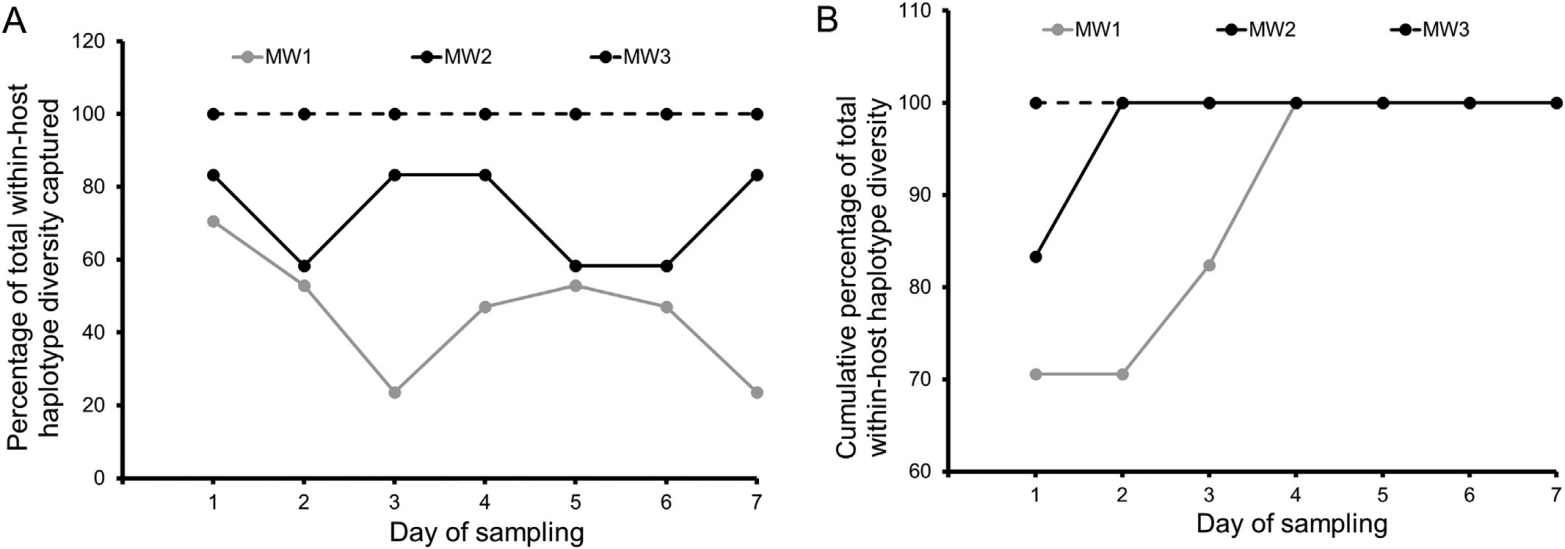
Temporal dynamics of co-infecting parasite haplotypes. *Panel A* shows the percentage of parasite haplotypes detected on each day of blood sampling compared with total parasite diversity within the patient. A single day sample often poorly captures within-host diversity. *Panel B* shows the cumulative percentage of parasite haplotypes detected on each day of sampling. Data show that repeat blood sampling is required to accurately capture within-host parasite ecology.

We observed rapid turnover of parasite haplotypes in patients MW1 and MW2 such that a single blood sample never captured the full array of parasite genotypes present in these individuals (Figs. 2, 3A and Supplementary Fig. S1). However, in patient MW3, a full array of parasite haplotypes observed on the first day of blood sampling continued to be detected on all the subsequent days of follow up (Fig. 2 and Supplementary Fig. S1). We plotted the cumulative percentage of parasite haplotypes detected on each day of blood sampling to determine sampling effort needed to capture all parasite haplotypes found in patients MW1 and MW2. While only two days of blood sampling were required to uncover the full spectrum of parasite haplotypes in patient MW1, four consecutive days of blood sampling were required to capture all parasite haplotypes detected in patient MW2 (Fig. 3B). Direct genotyping of serial blood samples from participants MW1, MW2 and MW3 without cloning yielded lower values of complexity of infection (multiplicity of infection or number of parasite haplotypes) than a combination of limiting dilution cloning of blood samples and SNP genotyping of parasite clones isolated (Supplementary Table S3).

### 3.5 Cloning efficiency

Within-host parasite diversity was efficiently captured in parasite clones isolated from each blood sample. Numbers of wells seeded with singly infected erythrocytes did not significantly differ (*p* > .05) from the 29 that is expected assuming Poisson distribution of parasite-positive erythrocytes in the 96-well plate.

### 3.6 Relatedness between parasite haplotypes

**Fig. 4:**
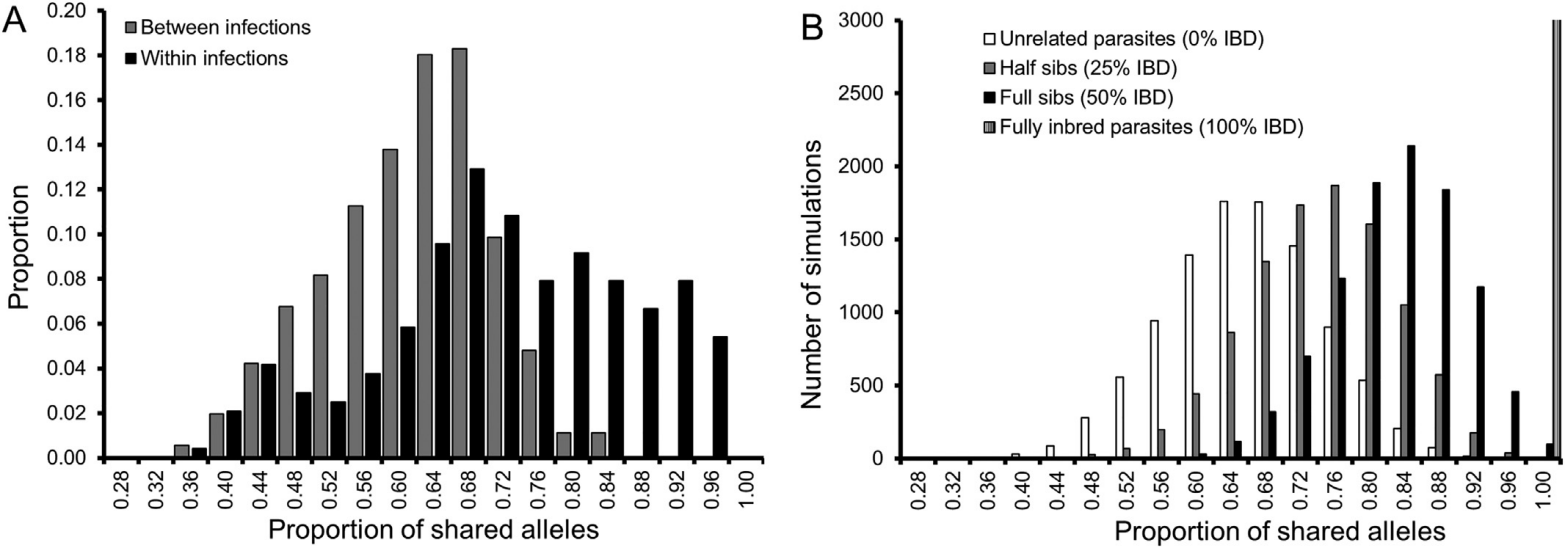
Relatedness of parasite haplotypes within asymptomatic infections. *Panel A* shows the observed frequency distribution of pairwise allele-sharing (*ps*) between parasite haplotypes recovered from the same or different infections. Grey bars denote comparisons between infections while black bars show within-infection comparisons. *Panel B* shows simulated allele-sharing (*ps*) distributions expected of parasites in the different classes of relatedness. We simulated *ps* expected of haploid parasites derived from the same inbred oocyst (100% identity-by-descent; IBD), from the same outcrossed oocysts (50% IBD), from two related oocysts (25% IBD) and from two unrelated oocysts (0% IBD). Upper and lower confidence limits for these *ps* distributions are shown in Table 1 and define thresholds for categorizing parasite haplotypes into different classes of relatedness.

Parasite haplotypes recovered from the same infection were more related than those from different infections (Fig. 4A). On average, parasite haplotypes within MHIs shared 70.8% of alleles (range: 33.3–95.8%) compared with 59.8% (range: 34.8–82.6%) between MHIs. Permutation analysis demonstrated that parasite haplotypes within MHIs are significantly more related than expected by chance (*p* < .0001). Analysis of both simulated and observed frequency distributions of pairwise allele-sharing confirmed significant differences in patterns of allele-sharing within and between patients (Fig. 4). All parasite haplotypes recovered from different patients showed pairwise allele-sharing expected of unrelated parasites. In contrast, 73.3% and 26.7% of parasite haplotypes within patients exhibited pairwise allele-sharing expected of unrelated and related parasites respectively. Of the 26.7% haplotype pairs deemed related, 7.5% were related at the half-sib level, 13.4% at the full-sib level while the rest (5.8%) were more related than full-sibs and are considered “extremely related parasites” (Nkhoma et al., 2012). Thresholds used to define the degree of parasite relatedness between parasite haplotypes recovered from the same or different infections are shown in Table 1.

**Table 1:** Simulated allele-sharing expected for parasites in different classes of relatedness.

Relatedness class	Mean	Lower 95% CL	Upper 95% CL
Unrelated	64.3	45.8	83.3
Half sibs	73.3	54.2	87.5
Full sibs	82.3	66.7	95.8
1st round inbred	91.0	79.2	100
2nd round inbred	95.6	87.5	100
3rd round inbred	97.7	91.7	100
4th round inbred	98.9	91.7	100
Fully inbred	100	100	100

## 4 Discussion

Several studies have focused on elucidating the population structure of malaria parasites in areas of variable endemicity as well as in locations where malaria control and elimination efforts are being intensified (Anderson et al., 2000; Manske et al., 2012; Mobegi et al., 2012; Nkhoma et al., 2012; Nkhoma et al., 2013; Mobegi et al., 2014; Delgado-Ratto et al., 2016; Friedrich et al., 2016; Hong et al., 2016; Fola et al., 2017; Fola et al., 2018). While important insights have emerged from such studies, our understanding of the within-host population structure of malaria parasites remains rudimentary. The full extent of within-host parasite diversity and the relative contribution of a variety of biological processes in generating diversity is not known especially in high transmission areas. This is primarily because we previously lacked high-throughput and high-resolution approaches for resolving infection complexity and relied on single snapshots of peripheral blood to measure within-host malaria parasite diversity. However, our understanding of the within-host population structure of malaria parasites has greatly advanced in recent years. This owes in part to the publication of whole genome sequences for the major malaria species that infect humans (Gardner et al., 2002; Carlton et al., 2008; Pain et al., 2008; Rutledge et al., 2017), and the development of next generation sequencing platforms and other high resolution approaches for assaying naturally occurring genetic variation (Su and Ferdig, 2002; Daniels et al., 2008; Juliano et al., 2010; Tan et al., 2011; Auburn et al., 2012; Manske et al., 2012; Mobegi et al., 2014). Our understanding of within-host malaria population structure has also greatly benefited from computational algorithms that make it feasible to handle, analyze and interpret large sets of parasite genetic data (Auburn et al., 2012; Assefa et al., 2014; O'Brien et al., 2016; Chang et al., 2017; Zhu et al., 2018). A better understanding of within-host malaria population structure is important because interactions between co-infecting parasite genotypes can profoundly influence the evolution of antimalarial drug resistance (Wargo et al., 2007; Antao and Hastings, 2011; Huijben et al., 2011), parasite virulence (Nowak and May, 1994; de Roode et al., 2005; Bell et al., 2006; Buckling and Brockhurst, 2008; Alizon, 2013; Alizon et al., 2013; Kada and Lion, 2015), malaria transmission (Taylor et al., 1997a; Read and Taylor, 2001), gametocyte sex ratio allocation (West et al., 2001; Reece et al., 2008; Pollitt et al., 2011), and malaria immunity (Cheesman et al., 2006). In this study, serial blood samples from 25 asymptomatic adults were analyzed to examine how accurately a single blood sample captures overall parasite diversity. Serial blood samples from three of the individuals were further analyzed to determine both the number and relatedness of co-infecting parasite haplotypes.

### 4.1 Does a single blood sample accurately capture within-host parasite diversity?

Our data show that a single peripheral blood sample taken from a malaria-infected individual often captures only a subset of parasite genotypes present within an individual. SNP genotyping demonstrated rapid turnover of parasite genotypes in serial blood samples from 88% (*n* = 22) of the asymptomatic infections studied with some parasite genotypes appearing while others disappearing from the peripheral blood within a matter of days (Fig. 2 and Supplementary Table S1). Msp-1 and msp-2 genotyping of serial samples from three of the 25 participants confirmed rapid genotype turnover in two individuals (MW1 and MW2) but no change in the parasite DNA fingerprint profile of participant MW3 (Supplementary Fig. S1). Further analysis of serial blood samples from the three participants provides further confirmation that parasite genotypes/haplotypes found in a single peripheral blood sample do not accurately reflect total parasite diversity within the host (Fig. 2) and should be viewed as only snapshots in time (Farnert, 2008). We provide several possible explanations for the rapid turnover of co-infecting parasite genotypes. First, this may reflect intra-host dynamics driven by host immunity. In previous longitudinal studies of asymptomatic infections, parasites bearing particular sets of antigenic markers were detectable for only short periods of time before being replaced by parasites with different sets of antigenic markers (Daubersies et al., 1996; Farnert et al., 1997; Farnert, 2008; Farnert et al., 2008). Second, rapid genotype turnover may be a form of antigenic variation used by co-infecting parasite genotypes to switch antigenic molecules expressed on the surface of parasite-infected erythrocytes. Akin to *var* gene switching (Recker et al., 2011), it could be an immune evasion mechanism to misdirect, confuse and evade the human immune system so that it fails to mount an effective response. Third, the rapid turnover of parasite genotypes could be due to sequestration of some co-infecting parasite genotypes in the deep tissue microvasculature (Montgomery et al., 2006). Co-infecting parasite genotypes with alternate 48-h replication cycles may appear and disappear from the peripheral blood depending on parasite stage and time the infected individual is sampled. Consistent with this reasoning, peripheral blood samples from malaria-infected individuals typically contain early asexual stages of the parasite (rings) but are often devoid of mature stages (trophozoites and schizonts). Sequestration of some co-infecting parasite genotypes could preclude their detection in the peripheral blood. Nonetheless, due to the 48-h periodicity of the *Plasmodium falciparum* asexual life cycle, sequestered parasite genotypes should emerge from sequestration sites after every 48 h and be detectable in the peripheral blood on alternate days of patient follow up as was observed in Papua New Guinea (Bruce et al., 2000) and in participant MW2 (Fig. 2 and Fig. 3B). The appearance of broods of parasites with alternating 48-h replication cycles is consistent with the finding that sequestered parasites are either genetically the same as those in the peripheral blood (Dembo et al., 2006; Waltmann et al., 2018) or constitute a subset of those circulating in the peripheral blood (Montgomery et al., 2006; Milner Jr. et al., 2012). Fourth, rapid genotype turnover could be due to the accumulation of spontaneous point mutations during asexual replication of parasites in the human host. It is also possible that different sets of co-infecting parasite genotypes are recovered randomly in dilution cloning experiments. However, dilution cloning of two independent cultures of the first serial blood sample from participant MW1 yielded identical sets of parasite haplotypes. This indicates that major parasite haplotypes generally remain stable after culture adaptation. It also indicates that rapid genotype turnover is not a mere stochastic process driven by genotype survival in culture but a real biological phenomenon as demonstrated in previous studies (Daubersies et al., 1996; Farnert et al., 1997; Farnert, 2008; Farnert et al., 2008). Another potential explanation for rapid genotype turnover is that genotyping tools used to fingerprint malaria infections failed to detect minor parasite variants within complex mixtures of parasite haplotypes. To minimize potential bias in resolving the genetic diversity of parasites within blood samples, we used a high resolution 24-SNP Molecular Barcode Assay to fingerprint infections. In addition, we resolved the number and relatedness of parasite haplotypes in serial blood samples from three participants by cloning infections and genotyping parasite clones isolated using the 24-SNP Molecular Barcode Assay. This detailed analysis provides an important glimpse into within-host dynamics of naturally occurring and untreated *Plasmodium falciparum* infections. Our finding that parasite genotypes recovered from a single peripheral blood sample may not fully account for total in vivo parasite diversity has important implications for understanding phenotype-genotype relationships. In most cases, such relationships are inferred from analyzing a single peripheral blood sample. If a single blood sample contains only a subset of parasite genotypes conferring the phenotype, incorrect parasite genotypes could be assigned to the observed phenotypes. For example, characterization of parasites, which cause cerebral malaria (CM), is of longstanding interest to the malaria research community (Montgomery et al., 2006; Seydel et al., 2006; Milner Jr. et al., 2012). However, there is concern that single snapshots of peripheral blood from CM patients may fail to capture parasites, which are sequestered in the brain, and responsible for CM pathology (Seydel et al., 2006; Milner Jr. et al., 2012). Repeat sampling of peripheral blood from CM patients may allow capture of previously undetected and sequestered parasite genotypes responsible for CM pathology. In contrast, if a measured phenotype is the average of each of the phenotypes of the different co-infecting parasite genotypes, we might expect the most abundant parasite genotype to exert a predominant effect on the overall phenotype. In this case, a single snapshot of peripheral blood may accurately resolve the relationship between phenotype and genotype because parasites that are predominantly sampled and phenotyped will be the same as those genotyped. For example, it is standard practice in antimalarial drug trials to examine associations between treatment outcome and parasite genotype (Djimde et al., 2001; Ariey et al., 2014; Amato et al., 2017), and to determine if episodes of parasitaemia persisting following therapy are due to treatment failure or re-infections. Because a single blood sample obtained at the time a patient fails treatment will contain parasites responsible for treatment failure, this single sample will adequately resolve the relationship between treatment outcome and parasite genotype. In contrast, if a co-infecting parasite genotype not detected prior to drug treatment is the only genotype sampled at the time the recurrent episode of parasitaemia occurs, persisting parasitaemia could be construed as a re-infection instead of treatment failure.The finding that a single blood sample may not accurately capture within-host parasite diversity underscores the importance of repeat blood sampling to resolve within-host parasite ecology. However, this may only be possible in asymptomatic adults because they can be left untreated for a short duration of time to study the natural course of infections. This is because asymptomatic adults have a lower risk of developing severe anaemia or other forms of life-threatening complicated malaria if left untreated. Repeat sampling of peripheral blood from drug-treated symptomatic individuals will fail to accurately capture naturally occurring patterns of diversity because drug treatment may clear some co-infecting parasite genotypes. Nonetheless, repeat sampling of drug-treated individuals could help identify minority drug-resistant or slow clearing parasite haplotypes within mixtures of predominantly susceptible or fast clearing parasite haplotypes (Mideo et al., 2016). This could be particularly useful for detecting minority drug-resistant or slow clearing parasites early on in resistance development. We therefore recommend that a longitudinal set of blood samples be taken to accurately capture within-host parasite ecology. However, for every study, the benefits of repeat blood sampling must be weighed against the cost of such blood draws and the risks including potential discomfort to study participants.

### 4.2 Complexity of asymptomatic malaria infections in adults from a high transmission area

Previous population surveys revealed important insights into within-host ecology of genetically diverse symptomatic malaria infections from Malawi (Juliano et al., 2010; Bailey et al., 2012; Nkhoma et al., 2012; Nair et al., 2014; Trevino et al., 2017). This study provides an important glimpse into within-host parasite diversity in asymptomatic malaria infections. We found that 19 of the 25 individuals sampled (76%) carried multiple parasite genotypes at baseline (Supplementary Table S1). Carriage of multiple parasite genotypes by a single human host is a common feature of both *P. falciparum* and *P. vivax* infections (Read and Taylor, 2001; Mobegi et al., 2012; Nkhoma et al., 2012; Delgado-Ratto et al., 2016; Friedrich et al., 2016; Fola et al., 2017; Fola et al., 2018). *Plasmodium vivax* is known to exhibit greater complexity of infection and higher rates of polyclonality than *P. falciparum* in locations where both species are sympatric (Pacheco et al., 2016; Fola et al., 2017). Therefore, interactions between co-infecting parasite genotypes can drive the evolution of biomedically important traits in both species. Detailed analysis of serial blood samples from three asymptomatic individuals demonstrated that they carried an average of 12 parasite haplotypes per person (Fig. 2). The number of parasite haplotypes observed matches some recent empirical estimates from Malawi (Nkhoma et al., 2012; Trevino et al., 2017). Direct genotyping of crude infections without dilution cloning underestimated within-host host parasite diversity (Supplementary Table S3) presumably because parasite haplotypes cannot be accurately reconstructed. Single time-point sampling of peripheral blood also poorly captured an infection and often under-sampled within-host parasite ecology (Fig. 2 and Fig. 3). Repeat sampling of individual patients uncovered much more diversity. However, a preponderance of sub-microscopic parasitaemias in asymptomatic infections is the major obstacle to obtaining a comprehensive and an unbiased assessment of parasite ecology in these infections. Because all infections analyzed in this study had sub-microscopic parasitaemias, they were culture-adapted prior to cloning to enrich them for malaria parasites. While culture adaptation combined with dilution cloning provided important insights into within-host parasite ecology, this approach may select for only parasite haplotypes that grow well in culture. Preferential selection of fast-growing parasite haplotypes would preclude characterization of parasite variants that grow poorly in culture or those that are outcompeted during culture. Parasite enrichment using culture-free methods (Trang et al., 2004; Tangchaikeeree et al., 2013) may be required to minimize potential bias in resolving within-host parasite ecology of low-density infections. Recent improvements in targeted capture of parasite-infected erythrocytes from complex mixtures and single cell sequencing (Trevino et al., 2017), coupled with repeat blood sampling should aid the resolution of within-host ecology of asymptomatic infections at unprecedented depth and scale.

### 4.3 Patterns of relatedness between co-infecting parasite haplotypes

Our data reveal a complex within-host relatedness structure with patterns of parasite co-transmission superimposed on those generated by superinfection. Based on simulated allele-sharing distributions expected for parasites in different classes of relatedness (Fig. 4B), ~70% of parasite haplotypes recovered within patients MW1, MW2 and MW3 were unrelated (proportion of shared alleles, ps < 83.3%; Table 1) while the rest were related at the half-sib level or greater. While these data provide important insights into within-host parasite relatedness structure, we are underpowered to accurately estimate the rates of both superinfection and parasite co-transmission in this setting. Akin to previous findings (Nkhoma et al., 2012; Nair et al., 2014; Trevino et al., 2017; Wong et al., 2017), our data demonstrate that a simple superinfection model in which co-infecting parasite haplotypes are assumed to be unrelated cannot fully explain patterns of parasite relatedness within individual hosts. There is greater role for parasite co-transmission in generating within-host parasite diversity than previously assumed in areas of intense malaria transmission. The inference made from genotyping human malaria infections that there is considerable parasite inbreeding even in high transmission areas is consistent with previous observations made from genotyping malaria infections in African mosquitoes (Annan et al., 2007). We found that approximately 30% of co-infecting parasite haplotypes are related, sharing 20 out of the 24 SNPs genotyped (ps > 0.833) (Table 1). This finding violates the key assumption of independence often made in studies and models of antimalarial drug resistance (Antao and Hastings, 2011), malaria transmission (Taylor et al., 1997b; Read and Taylor, 2001), parasite virulence (Nowak and May, 1994; Buckling and Brockhurst, 2008; Alizon et al., 2013; Kada and Lion, 2015) and sex ratio allocation (Reece et al., 2008; Pollitt et al., 2011). The central assumption made in these studies and models is that multiple-haplotype infections (MHIs) exclusively contain unrelated and independent parasite haplotypes randomly sampled from the local parasite population. Future studies need to account for the fact that parasite haplotypes within MHIs are not always unrelated and independent of each other. As in previous findings (Nkhoma et al., 2012), some co-infecting parasite haplotypes showed greater allele-sharing than full-sib parasites. These constituted 5.8% of related parasite haplotypes. Serial transmission, in which co-infecting parasite haplotypes are transmitted between multiple human hosts as intact units without being broken apart by recombination, can generate such highly related parasites (Nkhoma et al., 2012). It does so by progressively purging genetic variation similar to repeated inbreeding of laboratory model organisms such as mice (Broman, 2005).

Is it possible that some of the unrelated parasite haplotypes recovered within individual patients were recombinants inoculated through single mosquito bites but which subsequently diversified following a random mutation process in culture? To examine this possibility, we estimated the likelihood of our mutational target accumulating four point mutations over the 21 generations (42 days) it took to derive and expand individual parasite clones. We made two critical assumptions to achieve this. First, because the exact size of the target region bearing four divergent SNPs that distinguish related and related parasite haplotypes is unknown, we assumed that the 24 SNPs that we genotyped span a 24-kb genome segment since *P. falciparum* has a SNP density of ~ one SNP per kb (Mu et al., 2002). Second, we assumed that in *P. falciparum*, the rate of acquiring spontaneous point mutations is 1.0 × 10^−9^ to 4.6 × 10^−9^ per base pair per generation (Paget-McNicol and Saul, 2001; Bopp et al., 2013) and is the same for our mutational target. Based on these assumptions, we determined that there is probability of 0.0001 to 0.0005 for a 24-kb genome segment to acquire four point mutations over the timeframe observed. We therefore exclude the notion that four SNPs, which are divergent between related and unrelated parasite haplotypes, arose from a spontaneous mutation process during culture. However, because we do not know the age of these asymptomatic infections, we cannot rule out the possibility that mutation-driven diversification of co-transmitted parasite haplotypes occurred in individual hosts before infections were sampled. In addition, we cannot rule out the possibility that some of the unrelated parasite haplotypes were co-transmitted to individual human hosts via single mosquito bites. The observation that each of the three extensively studied individuals contained both related and unrelated parasite haplotypes renders some support for this notion. If a mosquito takes independent bloodmeals only a few days apart, it may be superinfected with unrelated parasite haplotypes. Subsequent inoculation of unrelated parasite haplotypes from a superinfected mosquito would yield patterns of parasite relatedness reminiscent of classical superinfection where unrelated parasites are inoculated into a single human host from sequential mosquito bites.

### 4.4 Implications for malaria parasite biology

(i) *Evolution of parasite virulence, drug resistance and malaria transmission*

The observation that a majority (~70%) of co-infecting parasite haplotypes were unrelated while the rest were related has important implications for understanding parasite virulence, drug resistance and malaria transmission. Within-host competition between unrelated parasite haplotypes selects for higher virulence, which increases both parasite virulence and transmission to the host (Taylor et al., 1997b; Alizon, 2013; Alizon et al., 2013). Within-host competition between drug-sensitive and drug-resistant parasite haplotypes also selects for increased drug resistance due to competitive release of drug-resistant parasites in the presence of drug treatment (Wargo et al., 2007; Huijben et al., 2011). While some studies have demonstrated a clear positive relationship between MHIs and parasite virulence to the host (de Roode et al., 2005; Bell et al., 2006), others have not (Abkallo et al., 2015). These discrepant findings could be explained by differences in the degree of relatedness between co-infecting parasite haplotypes. Consistent with this reasoning, a previous study in Colombia observed a strong association between MHIs and the severity of *P. vivax* but not *P. falciparum* malaria infections (Pacheco et al., 2016). Contrasting patterns between *P. falciparum* and *P. vivax* infections were attributed to the presence of distantly related parasite haplotypes in the latter (Pacheco et al., 2016). Similarly, increased parasite virulence in mice infected with multiple parasite strains may be explained by the fact that rodent malaria experimental systems are often constructed using mixtures of genetically unrelated parasites. Inconsistent relationships between MHIs and malaria severity could be reconciled by adjusting for parasite relatedness within infections. We would expect the magnitude of within-host competition between co-infecting parasite haplotypes to be reduced in individuals infected with related parasite haplotypes compared to those infected with distantly related parasite haplotypes. Increased within-host parasite relatedness would select for a lower level of parasite virulence as predicted from modelling the impact of parasite co-transmission on virulence evolution (Alizon, 2013). In addition, we would expect increased relatedness between co-infecting parasite haplotypes to reduce the level of competitive release (Wargo et al., 2007; Huijben et al., 2010; Huijben et al., 2011) and retard the spread of antimalarial drug resistance akin to the effect of less aggressive chemotherapy in rodent malaria experimental systems (Huijben et al., 2010).

(ii) *Evolution of gametocyte sex ratios and parasite virulence*

*Plasmodium* parasites are known to facultatively alter their gametocyte sex ratios in the presence of competing parasites and in response to resource availability such that they trade off investment in sexual transmissible stages relative to the asexual replicative stages to maximize their within-host competitive ability (Reece et al., 2008; Pollitt et al., 2011). A parasite haplotype will therefore reduce its investment in sexual stages in the presence of unrelated conspecifics because investment in sexual stages may jeopardize its within-host survival. Consequently, a parasite haplotype that shifts away from investment in gametocytes will become more virulent (Mideo and Day, 2008; Pollitt et al., 2011). Because ~70% of parasite haplotypes in three extensively sampled individuals were unrelated and subject to intense within-host competition, we would expect reduced investment in gametocyte production. This would result in gametocyte sex ratios that are less female-biased (Reece et al., 2008). In contrast, increased relatedness between co-infecting parasite haplotypes is expected to favour female-biased sex ratios and a lower level of virulence (Nkhoma et al., 2012). Reproductive restraint probably explains why in naturally infected human hosts, densities of sexual transmissible stages of the parasite tend to be orders of magnitude lower than those of asexual replicative stages (Taylor and Read, 1997). It ensures within-host survival of a parasite haplotype that could have been decimated by within-host competitive suppression.

## 5 Conclusion

In summary, our results demonstrate that a single peripheral blood sample taken from a malaria-infected individual often poorly captures within-host parasite ecology. These data reinforce previous findings showing that parasites in a single peripheral blood sample poorly track overall parasite diversity and should be considered as only snapshots in time. These findings underscore the importance of repeated blood sampling to accurately capture within-host parasite ecology. Our findings also demonstrate a critical role for both superinfection and parasite co-transmission in generating within-host parasite diversity. Taken together, these findings have important implications for understanding malaria transmission dynamics, the evolution of parasite virulence, drug resistance and sex ratio allocation.

The following are the supplementary data related to this article.

Table S1 SNP genotyping of asymptomatic malaria infections. Serial samples from each research participant were genotyped at 24 highly polymorphic SNPs as described previously (Daniels et al., 2008). The name of each SNP consists of the chromosome on which it is found and its position on the chromosome as annotated in PlasmoDB version 5.0. For example, SNP 1 Pf_01_000130573 describes a C/T SNP located on chromosome number 1 and position 000130573 of the P. falciparum genome. "–" shows a genotyping failure and N/A = not applicable. No. of heterozygous SNPs refers to the number of loci that carry both SNP alleles. Clonality refers to the genetic complexity of malaria parasites in an infection or sample i.e. whether the infection or sample contains multiple parasite haplotypes and is multiclonal (M) or a single parasite haplotype (S) and is monoclonal. Serial samples from the same study participant are shown using the same color scheme. Highlighted in red are SNP data for laboratory control parasites. Serial blood samples from the same individual were categorized as exhibiting rapid genotype turnover if they harbored different alleles at one or more loci or else if some serial samples carried two or more heterozygous (mixed) SNPs at loci that were monomorphic in other serial samples. These data demonstrate marked changes in the genetic composition of asymptomatic infections over seven consecutive days of blood sampling. These data add to the growing weight of evidence demonstrating that a single malaria-infected blood sample may not accurately capture overall parasite diversity within the host because it may only capture snapshots of time within a complex mixture of parasite haplotypes. Table S1

Table S2 SNP genotyping of parasite clones. Parasite clones isolated from all serial blood samples were genotyped at 24 highly polymorphic single nucleotide polymorphisms (SNPs) as described previously (Daniels et al., 2008).The name of each SNP consists of the chromosome on which it is found and its position on the chromosome as annotated in PlasmoDB version 5.0. For example, SNP 1 Pf_01_000130573 is a C/T SNP located on chromosome number 1 and position 000130573 of the P. falciparum genome. A parasite haplotype refers to a set of SNP alleles, which are inherited together as a unit. Clonality denotes the genetic complexity of a sample i.e. whether the sample contains multiple parasite genotypes (M) or a single parasite genotype (S). "–" shows a genotyping failure result and N/A = not applicable. No. of heterozygous SNPs refers to the number of loci that carry both SNP alleles. Parasite clones isolated from same individual are shown in blue, green or yellow. Highlighted in red are SNP genotype data for laboratory control parasites. These data show a much higher genetic diversity of parasites in asymptomatic malaria infections than previously assumed. Table S2

Table S3 Complexity of infection estimated from three different methods. Complexity of infection (COI) is an estimate of the number of parasite haplotypes present in a blood sample or infection. COI was estimated using three different methods: MSP genotyping, SNP genotyping and Cloning plus SNP genotyping. The latter gave higher COI values compared with direct SNP or msp1 and msp2 genotyping. This is probably because cloning and SNP genotyping unambiguously determines actual parasite haplotypes present in the infection/blood sample. These data suggest that asymptomatic infections sampled from adults residing in an area of intense malaria transmission exhibit far greater within-host parasite genetic diversity than was previously assumed. Table S3

Fig. S1 Msp-1 and msp-2 genotyping of serial blood samples from participants MW1, MW2 and MW3. Panels S1A to S1C show genotyping results for the MAD20, K1 and R033 msp-1 allelic variants while panels S1D and S1E show msp-2 genotype data for the 3D7/IC and FC27 allelic types respectively. Some serial blood samples from participants MW1 and MW2 show different parasite DNA fingerprints but all serial samples from participant MW3 have identical parasite DNA fingerprint profiles. These data show that a single blood often captures only a subset of parasite genotypes in an infection. Fig. S1
