# Supplementary material for: Intra-host dynamics of co-infecting parasite genotypes in asymptomatic malaria patients
Source: Infect Genet Evol. 2018 Nov;65:414–24. doi: 10.1016/j.meegid.2018.08.018 (PMC6219893; doi:10.1016/j.meegid.2018.08.018)
Supplement: Fig. S1 — Msp-1 and msp-2 genotyping of serial blood samples from participants MW1, MW2 and MW3. Panels S1A to S1C show genotyping results for the MAD20, K1 and R033 msp-1 allelic variants while panels S1D and S1E show msp-2 genotype data for the 3D7/IC and FC27 allelic types respectively. Some serial blood samples from participants MW1 and MW2 show different parasite DNA fingerprints but all serial samples from participant MW3 have identical parasite DNA fingerprint profiles. These data show that a single blood often captures only a subset of parasite genotypes in an infection. [file mmc4.pdf]

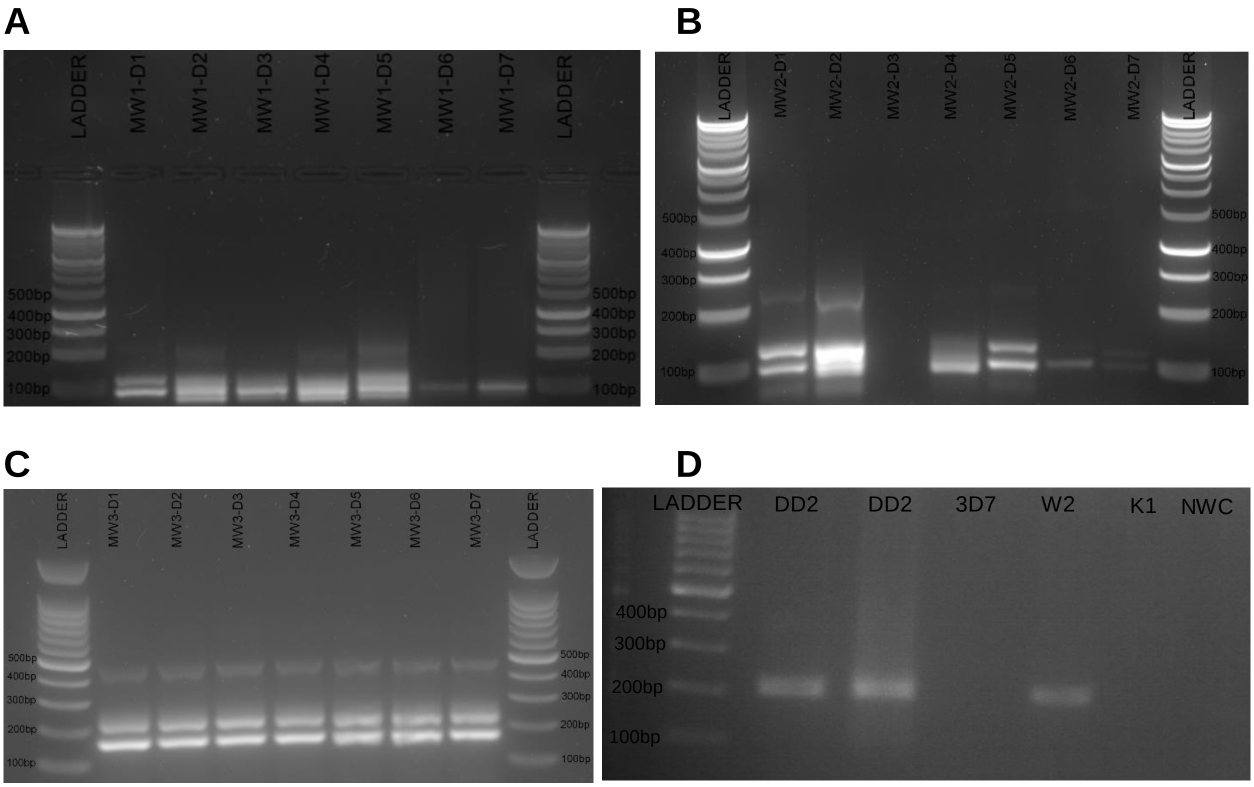

Figure S1A. Genotyping of the MAD20 msp-1 locus. Serial blood samples from participants MW1, MW2 and MW3 were genotyped at the MAD20 msp-1 locus. Panels A, B and C show genotyping results for serial blood samples from participants MW1, MW2 and MW3 respectively while panel D shows MAD20 genotype data for *Plasmodium falciparum laboratory* controls, DD2, 3D7, W2 and K1. NWC represents a negative water control; D1 to D7 are the seven days of blood sampling from each participant. Some serial blood samples from participants MW1 and MW2 show different parasite DNA fingerprints but all serial samples from participant MW3 have identical parasite DNA fingerprint profiles. These data show that a single blood often captures only a subset of parasite genotypes in an infection.

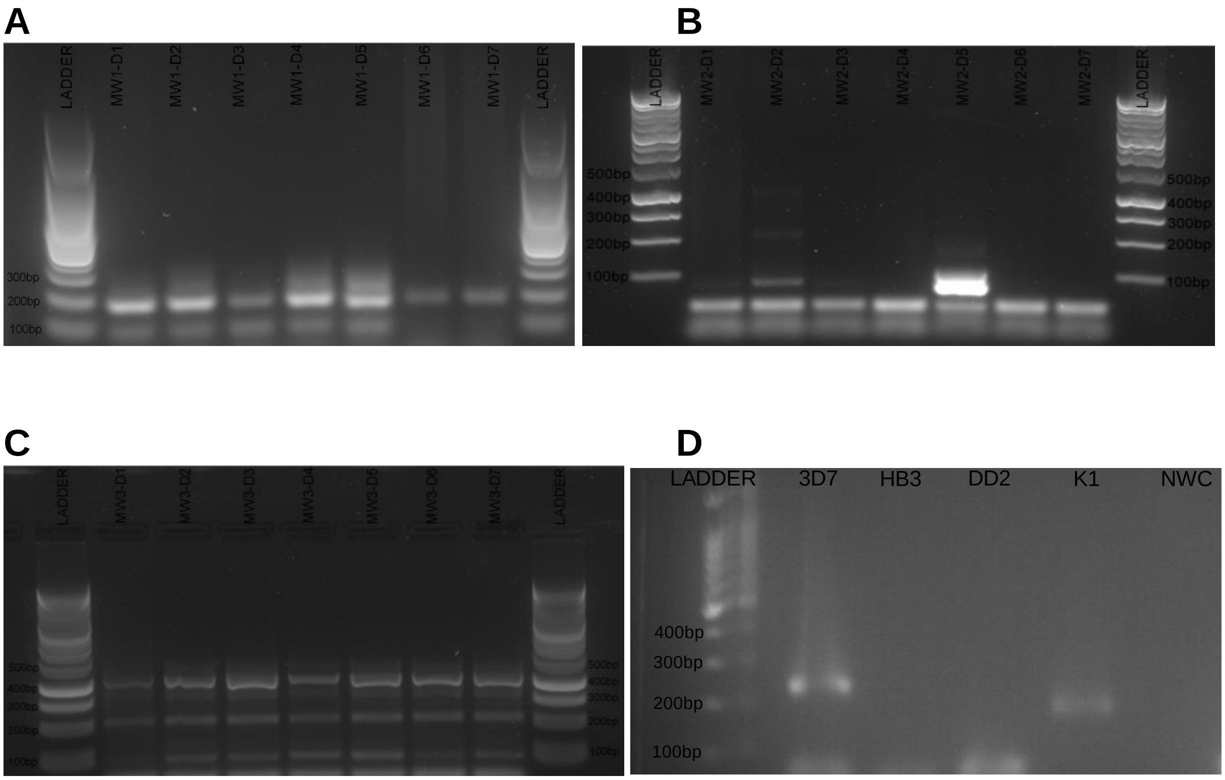

Figure S1B. Genotyping of the K1 msp-1 locus. Serial blood samples from participants MW1, MW2 and MW3 were genotyped at the K1 msp-1 locus. Panels A, B and C show genotyping results for serial blood samples from participants MW1, MW2 and MW3 respectively while panel D shows K1 genotype data for *Plasmodium falciparum laboratory* controls, 3D7, HB3, DD2 and K1. NWC represents a negative water control; D1 to D7 denote the seven days of blood sampling from each participant. Serial blood samples from participants MW1 and MW2 exhibit different parasite DNA fingerprint profiles on different days of sampling but those from participant MW3 show identical parasite DNA fingerprints on all the seven days of sampling. These data show that a single blood often captures only a subset of parasite genotypes in an infection.

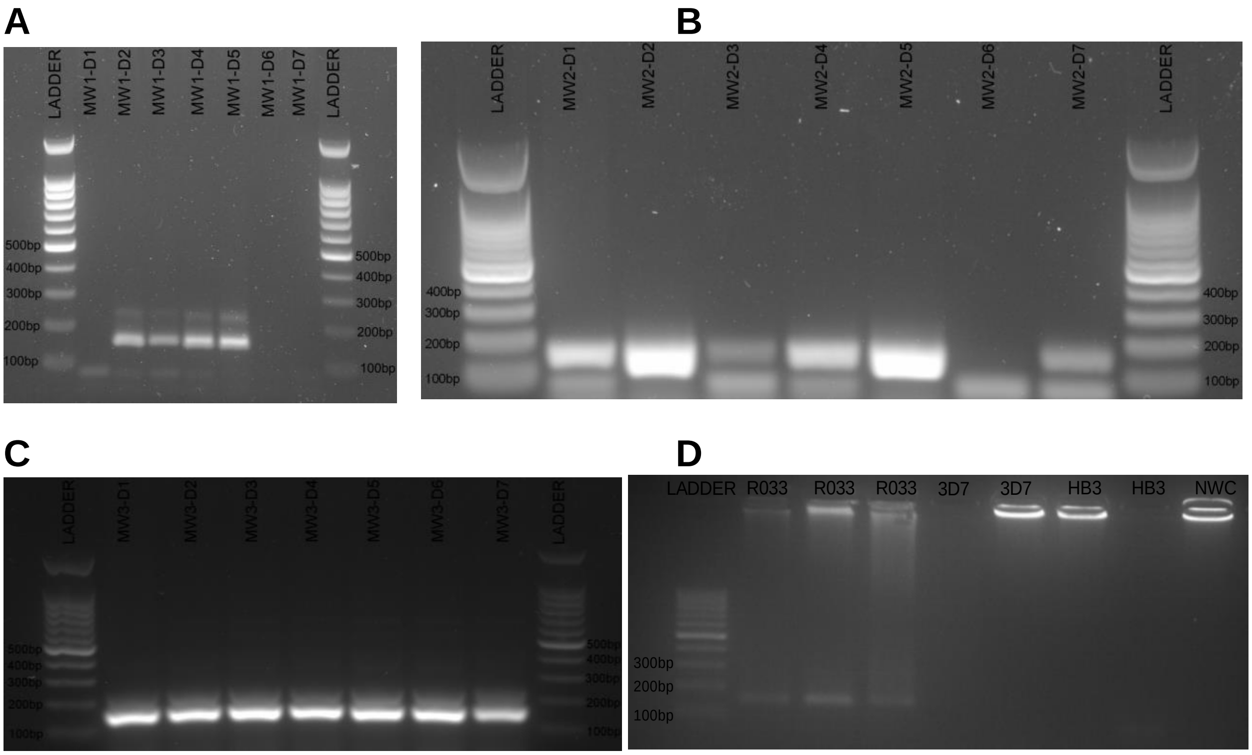

Figure S1C. Genotyping of the R033 msp-1 locus. Serial blood samples from participants MW1, MW2 and MW3 were genotyped at the R033 msp-1 locus. Panels A, B and C show genotyping results for serial blood samples from participants MW1, MW2 and MW3 respectively while panel D shows R033 genotype data for *Plasmodium falciparum laboratory* controls, R033, 3D7 and HB3. NWC represents a negative water control; D1 to D7 are the seven days of blood sampling from each participant. Some serial blood samples from participants MW1 and MW2 show different parasite DNA fingerprints but all serial samples from participant MW3 have identical parasite DNA fingerprint profiles. These data show that a single blood often captures only a subset of parasite genotypes in an infection.

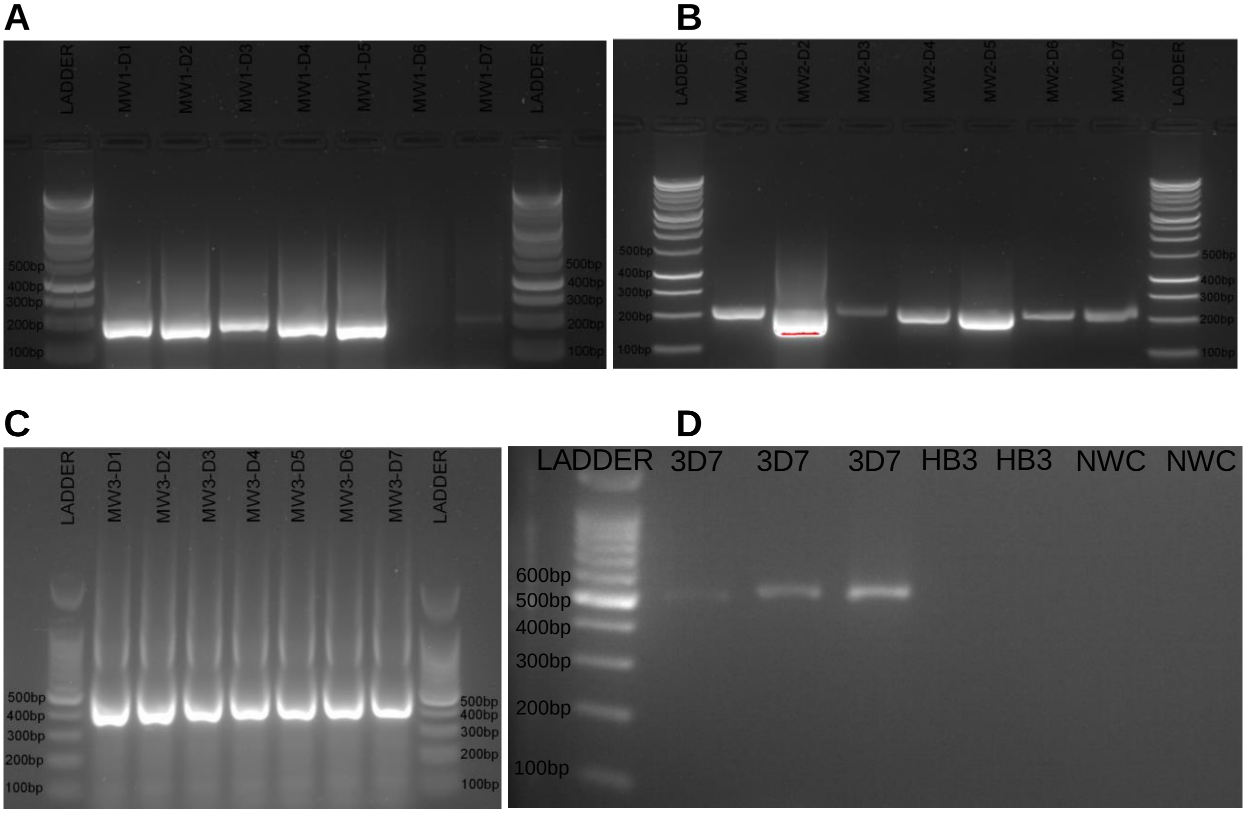

Figure S1D. Genotyping of the 3D7/IC msp-2 locus. Serial blood samples from participants MW1, MW2 and MW3 were genotyped at the 3D7/IC msp-2 locus. Panels A, B and C show genotyping results for serial blood samples from participants MW1, MW2 and MW3 respectively while panel D shows genotype data for *Plasmodium falciparum laboratory* controls, 3D7 and HB3. NWC represents a negative water control; D1 to D7 are the seven days of blood sampling from each participant. Some serial blood samples from participants MW1 and MW2 show different parasite DNA fingerprints but all serial samples from participant MW3 have identical parasite DNA fingerprint profiles. These data show that a single blood often captures only a subset of parasite genotypes in an infection.

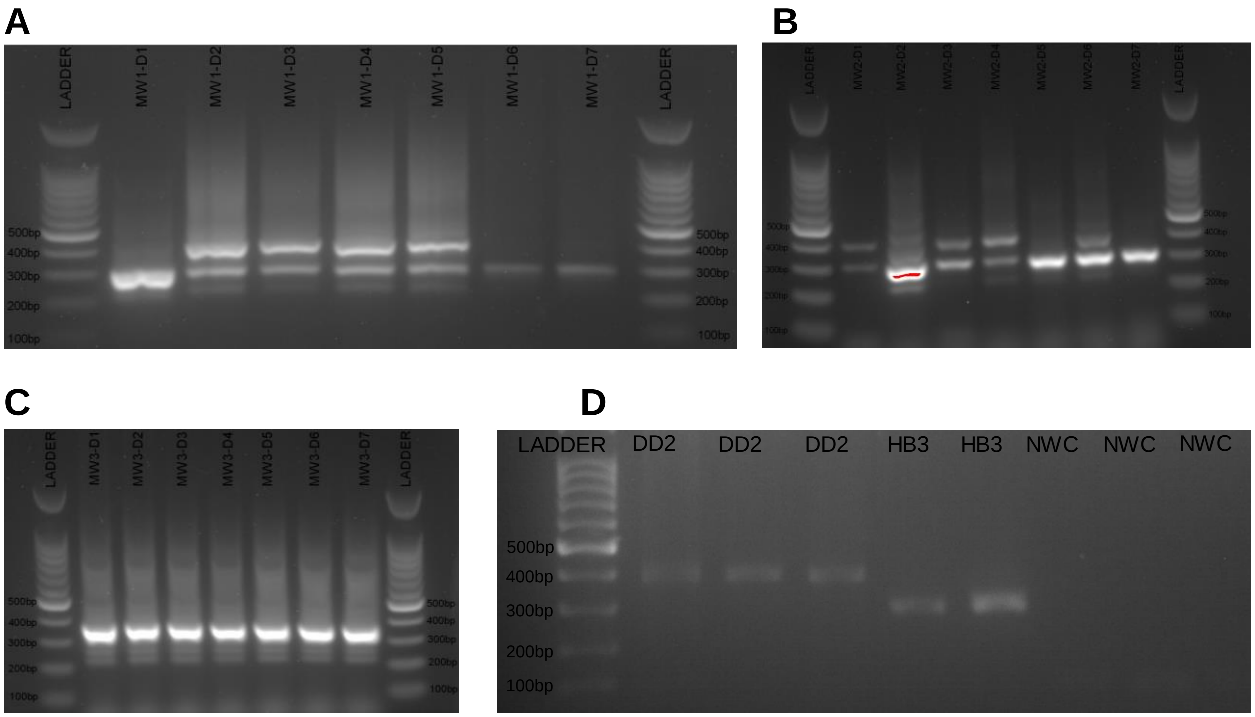

Figure S1E. Genotyping of the FC27 msp-2 locus. Serial blood samples from participants MW1, MW2 and MW3 were genotyped at the FC27 msp-2 locus. Panels A, B and C show genotyping results for serial blood samples from participants MW1, MW2 and MW3 respectively while panel D shows genotype data for *Plasmodium falciparum laboratory* controls, DD2 and HB3. NWC represents a negative water control; D1 to D7 are the seven days of blood sampling from each participant. Some serial blood samples from participants MW1 and MW2 show different parasite DNA fingerprints but all serial samples from participant MW3 have identical parasite DNA fingerprint profiles. These data show that a single blood often captures only a subset of parasite genotypes in an infection.
